# The CAG repeat polymorphism in the *Androgen receptor* gene modifies the risk for hypospadias in Caucasians

**DOI:** 10.1186/1471-2350-13-109

**Published:** 2012-11-20

**Authors:** Tatjana Adamovic, Agneta Nordenskjöld

**Affiliations:** 1 Department of Women's and Children's Health and Center of Molecular Medicine (CMM), Karolinska Institutet, SE-171 76, Stockholm, Sweden; 2 Pediatric Surgery Clinic, Astrid Lindgren Children Hospital, Karolinska University Hospital, SE-171 76, Stockholm, Sweden

**Keywords:** Hypospadias, Androgen receptor gene, CAG repeats

## Abstract

**Background:**

Hypospadias is a birth defect of the urethra in males, and a milder form of 46,XY disorder of sexual development (DSD). The disease is characterized by a ventrally placed urinary opening due to a premature fetal arrest of the urethra development. Moreover, the *Androgen receptor* (*AR)* gene has an essential role in the hormone-dependent stage of sexual development. In addition, longer AR polyglutamine repeat lengths encoded by CAG repeats are associated with lower transcriptional activity *in vitro*. In the present study, we aimed at investigating the role of the CAG repeat length in the *AR* gene in hypospadias cases as compared to the controls. Our study included 211 hypospadias and 208 controls of Caucasian origin.

**Methods:**

We amplified the CAG repeat region with PCR, and calculated the difference in the mean CAG repeat length between the hypospadias and control group using the T-test for independent groups.

**Results:**

We detected a significant increase of the CAG repeat length in the hypospadias cases when compared to the controls (contrast estimate: 2.29, 95% Confidence Interval (1.73-2.84); p-value: 0.001). In addition, the odds ratios between the hypospadias and controls revealed that the hypospadias cases are two to 3 times as likely to have longer CAG repeats than a shorter length for each repeat length investigated.

**Conclusions:**

We have investigated the largest number of hypospadias cases with regards to the CAG repeat length, and we provide evidence that a higher number of the CAG repeat sequence in the *AR* gene have a clear effect on the risk of hypospadias in Caucasians.

## Background

The *Androgen receptor* (*AR*) gene is located on chromosome Xq12 and is involved in the generation of the male internal and external genitalia through the actions of testosterone and 5α-dihydrotestosterone (DHT) [1]. The product of this gene belongs to the nuclear receptor class, and is expressed in the developing human penis and urethra [2]. It affects the expression of androgen regulated genes critical for the development of the male sexual phenotype by recognizing the canonical androgen response elements (AREs) in the DNA once it has formed a complex with DHT or testosterone in the cytoplasm [3].

Mutations in the *AR* gene that severely impact the function of the receptor can cause the syndrome of partial or complete androgen insensitivity, and have also been observed in a few cases of isolated hypospadias [4-10]. The malformation of hypospadias is described as a birth defect of the urethra in males, and is considered to be a milder form of 46,XY disorder of sex development (DSD). It is characterized by a ventrally placed urinary opening in boys and affects 3 per 1000 males in Sweden since the beginning of the 1970s (data from the annual Swedish Malformation Registry). This disease is influenced both by genes and environmental factors, and about 10 % of these boys have a family history of hypospadias. Most cases with hypospadias are reported to be sporadic [11].

Hypospadias is usually diagnosed during physical examination of the newborns, with subsequent surgery in their first two years of life. Still, the molecular mechanisms behind this disease, particularly in the sporadic form, are largely unknown.

Since AR mediates important biological effect of testosterone and DHT, it is an obvious candidate in the development of hypospadias. Moreover, greater number of CAG repeats within the *AR* gene results in longer polyglutamine tracts in the AR, and cause the AR to have a reduced transcriptional activity and to associate with moderate to severe undermasculinized genitalia in XY males [12]. In addition, we previously performed a study in which we [13], and another independent group [14], assessed the CAG repeat length in a small number of cases with hypospadias. However, no association was found between the CAG repeat length and the investigated cases when compared to the controls. In the present study, we decided to investigate possible association of the CAG repeat length in the *AR* gene with the disease of hypospadias in a significantly larger patient material.

## Results

### The CAG repeat length in cases with hypospadias and in controls

**Figure 1:**
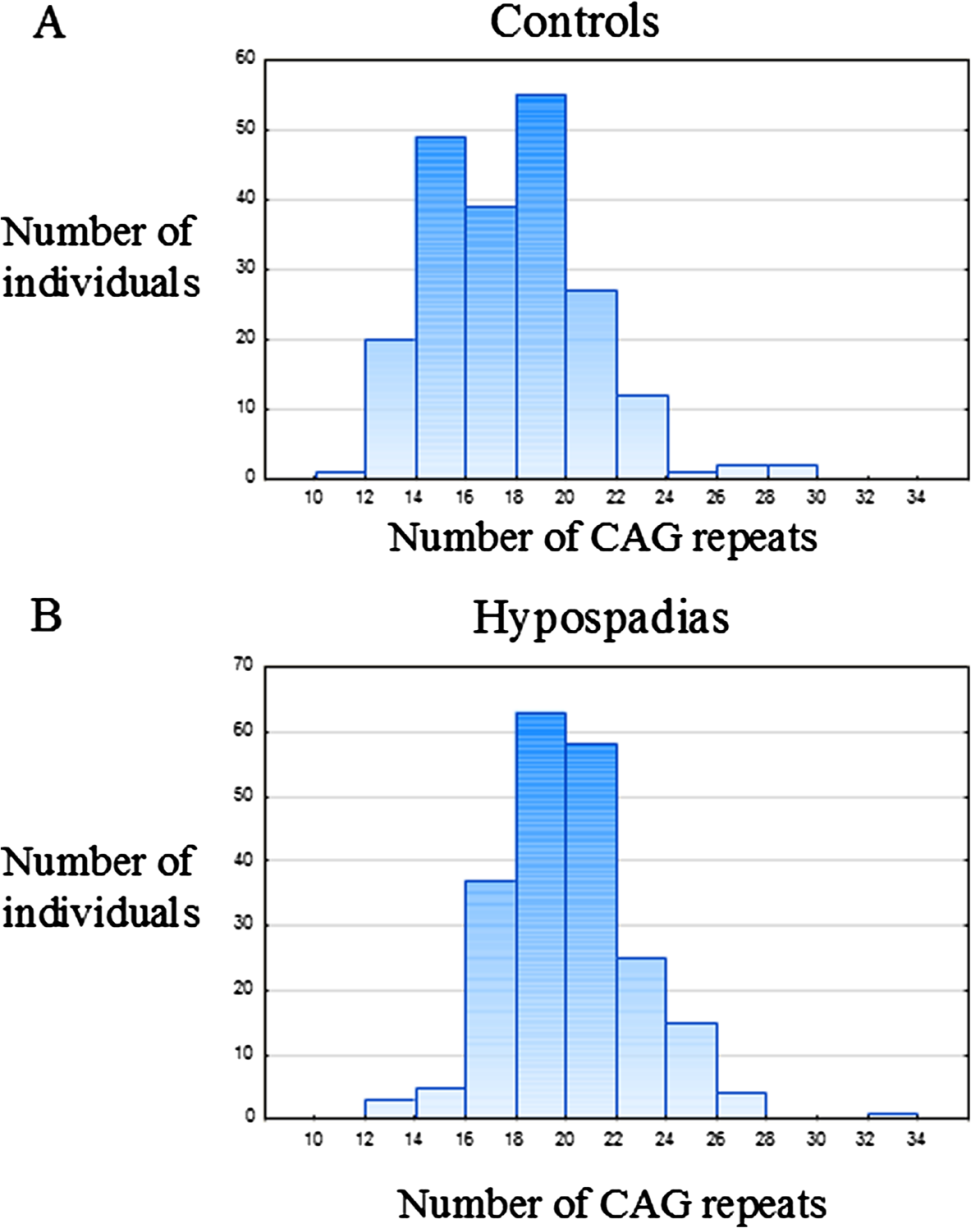
**Distribution of CAG repeat sizes in exon 1 of the *AR *gene in controls and in cases with hypospadias.**

We compared the number of CAG repeats in the *AR* gene in each case from the hypospadias group with the repeats in individuals from the control group. We first calculated the skewness for each group, and detected the CAG repeats to be normally distributed within each group (see Table 1; mean and median length are also presented). We next used the T-test for independent groups, and found a clear statistical difference in the mean CAG repeat length between the control and hypospadias group (p<0.001). Thus, the estimated mean CAG repeat length difference was calculated to 2.29 (CI, 1.73-2.84). We further detected a significant difference in the median CAG repeat length between the two groups using the Chi-square test (p<0.001). Figure 1 illustrates the distribution of the repeats, and clearly shows a greater frequency of the longer repeat tracts in the hypospadias group.

**Table 1:** Mean and median CAG repeat length in the hypospadias and control group

**Hypospadias**	**Cases (n)**	**Mean**	**Median**	**Minimum**	**Maximum**	**Lower quartile**	**Upper quartile**	**SD**	**Skewness**
Nr. of CAG repeats	211	**20.07**	**19.87**	13.13	32.07	18.22	21.73	2.76	0.56
**Controls**	Controls (n)	Mean	Median	Minimum	Maximum	Lower quartile	Upper quartile	SD	Skewness
Nr. of CAG repeats	208	**17.78**	**17.78**	11.00	29.77	15.62	19.41	3.00	0.76

*Abbreviations:* SD, standard deviation

Moreover, Odds Ratio (OR) was calculated between the hypospadias and the control group for a range of repeat sizes using logistic regression (see Table 2). A significant OR was detected for each repeat size investigated (Table 2).

**Table 2:** Odds Ratio (OR) analysis of the CAG repeat length in the hypospadias group as compared to the controls

**CAG nr.**	**Control %**	**Hypospadias %**	**OR**	**95% CI**
≤18	52.40	21.33		
≥18	48.56	78.67	3.91*	2.55-5.99
≥19	34.62	61.61	3.03*	2.03-4.51
≥20	21.63	48.82	3.45*	2.25-5.29
≥21	12.50	36.02	3.94*	2.39-6.48
≥22	8.65	21.33	2.86*	1.59-5.13
≥23	4.81	16.59	3.94*	1.89-8.18
≥24	2.40	9.48	4.25*	1.56-11.55
≥25	1.92	3.79	2.01*	0.60-6.78
≥26	1.92	2.37	1.24*	0.33-4.67

*Abbreviations:* OR, Odds Ratio; 95% CI, lower and upper 95% confidence intervall limit; *, significant p-value, i.e. < 0.05.

## Discussion

In the present investigation, we detected a significantly expanded CAG repeat length located in exon 1 of the *AR* gene in the largest number of examined Caucasian patients with hypospadias reported so far. Thus, our findings suggest a role of the CAG repeat polymorphism in the development of hypospadias.

Several pathways have up until now been implicated in male urethra development, and the strong focus of genetic research on the hormone-dependent stage of sexual development has revealed promising candidate genes, among which the *AR* gene is included. Expansion of the CAG repeat length in exon 1 has previously been reported to decrease AR transactivation function [15]. An even larger increase in the number of CAG repeats in the *AR* gene has further been detected in the rare X-linked neurodegenerative disorder, spinal and bulbar muscular atrophy (SBMA), or Kennedy’s disease [16], where the expanded repeats varied between 40–62, compared to 11–31, as seen in healthy individuals [17,18]. It has also been reported that other polymorphisms, such as longer GGN repeat lengths in the AR, increase the risk for penile hypospadias [13,19]. These latter reports, which include our previously published data [13], were not able to detect any association between the CAG repeat length and the disease of hypospadias, which may be due to the small sample size investigated. In our present study, we describe the investigation of a much larger patient material, in which we detect a significant difference of the CAG repeat length in the cases, yielding a markedly increased sequence length. Thus, the magnitude of the difference in mean CAG repeat length was estimated to 2.29. Moreover, the calculated ORs between the hypospadias cases and controls revealed that the hypospadias cases are two to 3 times as likely to have longer CAG repeats than a shorter length for each repeat length investigated. Hence, the event may cause a decrease in the AR transactivation function, as previously reported [12,15].

## Conclusions

Our analysis suggests a role of expanded CAG repeat length in the development of hypospadias. Based on our results, we propose that the CAG polymorphism modifies the risk of hypospadias in Caucasians.

## Methods

We investigated DNA from 211 cases with hypospadias. The cases composed of 33 patients with penoscrotal, scrotal or perineal phenotype, and 178 with glandular, coronar, mid or distal penil phenotype, as classified by A.N (pediatric surgeon at Karolinska University Hospital). The control group consisted of 208 healthy voluntary anonymous blood sample donors, all males, from the Karolinska University Hospital. The cases and controls were of Caucasian origin. Genomic DNA was extracted from either tissue harvested at surgery or from peripheral blood according to standard procedures reported elsewhere [20]. This study was approved by the Ethics Committee at Karolinska University Hospital. Informed written consent was obtained from all participants involved in the study.

### Tri glutamine-repeats investigation

The CAG repeat region located in the *AR* gene was PCR amplified and mixed with 500-Rox size standard, and electrophoresed on the ABI3730 Sequencer (Applied Biosystems). Primer and PCR amplification conditions have been reported previously [13]. In total, 211 hypospadias cases and 208 male controls were investigated. A difference in the mean CAG repeat length between the hypospadias and control group was tested using the T-test for independent groups, since the data was considered to be normally distributed. Furthermore, using Chi-Square test we calculated whether a significant difference in the median length could be detected. In addition we compared the two groups for a range of repeat sizes (Odds Ratio and Confidence Interval was calculated) using logistic regression. The statistical analysis was implemented using Statistica 10.0, StatSoft® (Inc. Tulsa OK, USA).

## Competing interests

The authors declare no competing interests.

## Authors’ contributions

TA and AN conceived and designed the study. TA and AN performed the PCR and statistical analysis. TA and AN interpreted the data and wrote the manuscript. All authors read and approved the final manuscript.

## Pre-publication history

The pre-publication history for this paper can be accessed here:

http://www.biomedcentral.com/1471-2350/13/109/prepub

## References

[B1] Siiteri PK Wilson JD Testosterone formation and metabolism during male sexual differentiation in the human embryo J Clin Endocrinol Metab 1974 38 113 125 10.1210/jcem-38-1-113 4809636

[B2] Kim KS Liu W Cunha GR Russell DW Huang H Shapiro E Baskin LS Expression of the androgen receptor and 5 alpha-reductase type 2 in the developing human fetal penis and urethra Cell Tissue Res 2002 307 145 153 10.1007/s004410100464 11845321

[B3] Mooradian AD Morley JE Korenman SG Biological actions of androgens Endocrine review 1987 8 1 28 10.1210/edrv-8-1-1 3549275

[B4] Hiort O Klauber G Cendron M Sinnecker GH Keim L Schwinger E Wolfe HJ Yandell DW Molecular characterization of the androgen receptor gene in boys with hypospadias Eur J Pediatr 1994 153 317 321 10.1007/BF01956409 8033918

[B5] Alléra A Herbst MA Griffin JE Wilson JD Schweikert HU McPhaul MJ Mutations of the androgen receptor coding sequence are infrequent in patients with isolated hypospadias J Clin Endocrinol Metab 1995 80 2697 2699 10.1210/jc.80.9.2697 7673412

[B6] Sutherland RW Wiener JS Hicks JP Marcelli M Gonzales ETJ Roth DR Lamb DJ Androgen receptor gene mutations are rarely associated with isolated penile hypospadias J Urol 1996 156 828 831 10.1016/S0022-5347(01)65830-0 8683794

[B7] Nordenskjöld A Friedman E Tapper-Persson M Söderhäll C Leviav A Svensson J Anvret M Screening for mutations in candidate genes for hypospadias Urol Res 1999 27 49 55 10.1007/s002400050088 10092153

[B8] Wang Y Li Q Xu J Liu Q Wang W Lin Y Ma F Che T Li S Shen Y Mutation analysis of five candidate genes in Chinese patients with hypospadias Eur J Hum Genet 2004 12 706 712 10.1038/sj.ejhg.5201232 15266301

[B9] Thai HT Kalbasi M Lagerstedt K Frisén L Kockum I Nordenskjöld A The valine allele of the V89L polymorphism in the 5-alpha-reductase gene confers a reduced risk for hypospadias J of Endocrinol and Metab 2005 90 6695 6698 10.1210/jc.2005-0446 16174723

[B10] Wong HY Hoogerbrugge JW Pang KL van Leeuwen M van Royen ME Molier M Berrevoets CA Dooijes D Dubbink HJ van de Wijngaart DJ A novel mutation F826L in the human androgen receptor in partial androgen insensitivity syndrome; increased NH2-/COOH-terminal domain interaction and TIF2 co-activation Mol Cell Biol 2008 24 69 78 10.1016/j.mce.2008.06.016 18656523

[B11] Fredell L Kockum I Hansson E Holmner S Lundquist L Läckgren G Pedersen J Stenberg A Westbacke G Nordenskjöld A Heredity of hypospadias and the significance of low birth weight J Urol 2002 167 1423 1427 10.1016/S0022-5347(05)65334-7 11832761

[B12] Lim HN Chen H McBride S Dunning AM Nixon RM Hughes IA Hawkins JR Longer polyglutamine tracts in the androgen receptor are associated with moderate to severe undermasculinized genitalia in XY males Hum Mol Genet 2000 22 829 834 10749991 10.1093/hmg/9.5.829

[B13] Aschim EL Nordenskjöld A Giwercman A Lundin KB Ruhayel Y Haugen TB Grotmol T Giwercman YL Linkage between cryptorchidism, hypospadias, and GGN repeat length in the androgen receptor gene J Clin Endocrinol Metab 2004 89 5105 5109 10.1210/jc.2004-0293 15472213

[B14] Muroya K Sasagawa I Suzuki Y Nakada T Ishii T Ogata T Hypospadias and the androgen receptor gene: mutation screening and CAG repeat length analysis Mol Hum Reprod 2001 7 409 413 10.1093/molehr/7.5.409 11331662

[B15] Chamberlain NL Driver ED Miesfeld RL The length and location of CAG trinucleotide repeats in the androgen receptor N-terminal domain affect transactivation function Nucleic Acids Res 1994 22 3181 3186 10.1093/nar/22.15.3181 8065934 PMC310294

[B16] La Spada AR Wilson EM Lubahn DB Harding AE Fischbeck KH Androgen receptor gene mutation in X-linked spinal and bulbar muscular atrophy Nature 1991 352 77 79 10.1038/352077a0 2062380

[B17] La Spada AR Roling D Harding AE Warner CL Spiegel R Hausmanowa-Petrusewicz I Yee WC Fischbeck KH Meiotic stability and genotype-phenoype correlation of the trinucleotide repeat in X-linked spinal and bulbar muscular atrophy Nat Genet 1992 2 301 304 10.1038/ng1292-301 1303283

[B18] Edwards CA Hammond HA Jin L Caskey CT Chakraborty R Genetic variation in five trimeric and tetrameric tandem repeat loci in four human population groups Genomics 1992 12 241 253 10.1016/0888-7543(92)90371-X 1740333

[B19] Radpour R Rezaee M Tavasoly A Solati S Saleki A Association of long polyglycine tracts (GGN repeats) in exon 1 of the androgen receptor gene with cryptorchidism and penile hypospadias in Iranian patients J Androl 2007 28 164 169 16957138 10.2164/jandrol.106.000927

[B20] Sambrook J Fritsch E Maniatis T Isolation of high molecular weight DNA from mammalian cells 1989 Molecular Cloning New York: Cold Spring Harbour Laboratory Press 14 23

